# Dissolution Kinetics in Plasma-Enhanced Nitric Acid Solvolysis of CFRCs

**DOI:** 10.3390/ma18184242

**Published:** 2025-09-10

**Authors:** Dimitrios Marinis, Ergina Farsari, Eleftherios Amanatides

**Affiliations:** Plasma Technology Laboratory, Department of Chemical Engineering, University of Patras, 26504 Patras, Greece; marinis@chemeng.upatras.gr (D.M.); efarsari@chemeng.upatras.gr (E.F.)

**Keywords:** recycling, CFRP, plasma solvolysis, nitric acid, carbon fiber, plasma in bubbles

## Abstract

The dissolution kinetics in conventional nitric acid and plasma-enhanced nitric acid solvolysis of composites were investigated. Unidirectional carbon fiber epoxy laminates originating from the scar of wind turbine blades were used for the study. The carbon fiber retrieval rate was experimentally determined as a function of dissolution time and composite mass. A kinetic model, which included disintegration of the polymer matrix and the mass transport of polymer fragments to the liquid phase, was implemented to investigate the main parameters that affect the dissolution rate. The plasma enhancement and the increase of the composite mass favor the carbon fiber retrieval rate, while process time slows down the matrix dissolution rate. The composite surface in contact with the liquid, solid-to-liquid volume ratio, solubility of the polymer matrix, and disintegration and mass transport rate coefficients have a significant effect on the dissolution rate, and the rate-limiting factors were revealed and analyzed.

## 1. Introduction

Carbon fiber-reinforced composites (CFRCs) have several applications in military, aerospace, and automotive industries due to their superior properties, such as strength, durability, high strength-to-weight ratios, and corrosion resistance [1,2]. Yet, the leftover material and defective products generated during the production of CFRCs as well as the waste products that reach end of life contain a large amount of high-performance carbon fibers (CFs). The scale of this composite waste has become increasingly critical, with global CFRC market demand reaching EUR 6.5 billion in 2024 and projected to expand to EUR 12.0 billion by 2030. Manufacturing processes generate approximately 30–40% of total composite materials as production scrap, including prepreg off-cuts, mis-molded parts, and trim waste, amounting to roughly 68 kilotons of composite waste annually. By 2025, Europe alone is projected to generate 683 kilotons of CFRC waste annually, while global projections indicate that aircraft and wind turbine industries will collectively produce 840 kilotons of CFRC waste annually by 2050. The aerospace sector contributes significantly to this burden, with approximately 12,000 aircraft worldwide expected to reach end-of-life status within the next two decades, not including newer CFRC-intensive models like the Boeing 787 and Airbus A350 XWB, which will enter waste streams after 25–30 years of service. Wind turbine blade decommissioning represents another substantial waste source, with Europe expected to handle 55–60 kilotons of end-of-life blade material by 2025. Current estimates suggest that landfill-stocked CFRC waste is worth approximately EUR 14.7 million in recoverable carbon fiber value, considering a EUR 10/kg market price for the recycled material. Global recycling capacity remains severely limited at less than 100 kilotons annually, meaning that over 583 kilotons of composite waste will follow a disposal path to landfills or incineration [3,4,5,6,7,8]. The retrieval of CFs from this waste is not an easy task, as CFRCs have a three-dimensional cross-linked network structure that cannot be re-melted and used for secondary molding [9,10]. Thus, CFRC recycling has become the subject of many studies, and the current methods for recovering carbon fibers from waste composites encompass mechanical, thermal, and chemical processes, with each one of them offering distinct advantages and facing specific limitations [11,12,13,14,15,16,17,18,19,20,21,22,23,24,25,26,27,28,29,30,31,32]. Mechanical methods such as shredding, milling, and grinding are relatively low-cost and straightforward to implement, but they severely damage fiber length and surface integrity, yielding short, fragmented fibers with reduced mechanical properties that restrict reuse to low-value applications [11]. Thermal processes can efficiently remove the polymer matrix to release fibers of moderate quality and allow resin recovery; however, they demand temperatures above 400 °C and releasing harmful gases such as CO_2_ and CO, thus requiring gas treatment and driving up operational costs [12,13]. Chemical recycling methods have attracted particular attention because high value-added chemicals and monomers can be recovered by the selective cleavage of the chemical bonds in solvent–catalyst systems. However, these methods rely on expensive solvents and catalysts while producing organic and inorganic by-products, and they often require complex separation and purification steps, which can limit economic viability and environmental performance [14,15,16,17,18,19,20,21,22,23,24,25,26,27,28,29,30,31,32].

Chemical recycling includes the low-temperature and the supercritical fluid dissociation method. Super/sub-critical fluids, like water or organic solvents, offer resin decomposition rates, allowing the recovery of high-quality fibers, but require high temperatures and pressures during operation, thus making scale-up challenging and energy-intensive [19,20,21,22]. Low-temperature chemical methods include the alcoholysis under alkaline catalysis [23,24,25,26] and wet oxidation methods [27,28,29], where a high-concentration acidic environment (nitric acid, acetic acid, peracetic acid, and hydrogen peroxide) is used for the dissolution of the composite. Both processes are rather slow; thus, different alternatives, which include electrochemical, plasma, and sonication enhancement, have been proposed [29,30,31,32]. The most important advantage of wet oxidation methods is their consistency when it comes to decomposing unknown types of CFRCs, which is a very common case for wastes that were produced 20 to 30 years before and have reached their end of life. Specifically, nitric acid emerges as the most effective oxidant, ensuring high-quality recovered fibers [27,28,32]. Despite its efficacy, the drawback lies in the extended decomposition times, ranging from 20 to 100 h, which significantly limits the throughput of the process and gives rise to environmental concerns due to the production of nitrogen oxides and liquid wastes [14,16,21,27,32].

Recently, it was shown that the dissolution rates in nitric acid solvolysis of composites can be enhanced by the implementation of atmospheric air and argon plasma in bubbles of nitric acid solution [32,33] while, at the same, time high-quality continuous CFs can be retrieved [34]. Previous results and life-cycle assessment analysis have also shown that the method is competitive to already-established technologies in terms of cost, capacity, and environmental impact [33,35]. In the current work, a detailed analysis of the dissolution mechanism and kinetics in the nitric acid and in the plasma-enhanced nitric acid solvolysis of CFRCs is presented. The main objective is the determination of the process parameters that can be dissolution rate-limiting factors. In this direction, the effects of composite mass and of treatment time on the CF retrieval rate were experimentally investigated. A simplified model of solid dissolution is used to reveal the main parameters that affect the dissolution process and explain the changes in the retrieval rates that were experimentally observed. The rate-limiting factors of solvolysis are presented, and possible ways to enhance dissolution rates are discussed.

## 2. Materials and Methods

### 2.1. Materials

In this work, cubic turbine blade scrap specimens were used. The CF composite parts originated from the spar of a 60 m long wind turbine blade, which was dismantled into several parts (shear, spar, core, shell) and cut into smaller pieces. The spar consisted of unidirectional glass and carbon fiber epoxy laminates and was cut with a circular saw into cubes of different sizes. In this study, only unidirectional carbon fiber epoxy laminates were used for recycling, and the edge of the cubes varied between 2.3 and 2.75 cm. Although no specific material properties were known, the density of the material was measured equal to 1.44 g/cm^3^, and the CF content in the composite was evaluated equal 67%. These values are typical for epoxy resin carbon fiber composites. For the plasma solvolysis process, concentrated nitric acid (65% *w*/*w*, Honeywell, Charlotte, NC, USA), Ar (99.999% NOVOGAS, Patras, Greece), and compressed atmospheric air were used, while acetone (2-propanone, wt/wt 99.8%, Fisher Chemical, Pittsburgh, PA, USA) was used for the resin residual removal.

### 2.2. Experimental Set-Up and Procedure

**Figure 1:**
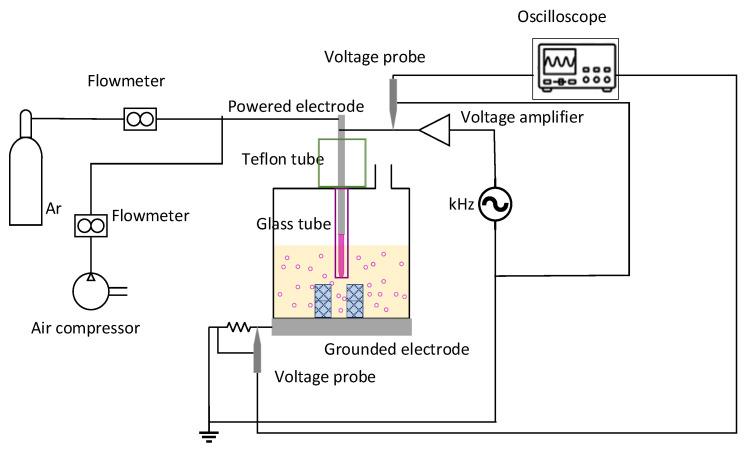
Plasma enhanced solvolysis reactor.

Figure 1 illustrates the experimental set-up. The reactor consists of a 250 cm^3^ glass container placed on a stainless-steel plate, which acts as the grounded electrode of the system. The powered electrode is a ¼’’ stainless steel tube and is surrounded by a dielectric Pyrex glass tube. The powered electrode is placed at a distance of 1 cm above the solution (concentrated HNO_3_) surface, while the glass tube is extended 6 cm inside the liquid.

The powered electrode was excited by a system comprising a high-frequency generator (30 kHz signal generator IGBT143, Martignoni Elettrotecnica, Vestone, Italy) and voltage amplifier (IGBT163, Martignoni Elettrotecnica, Vestone, Italy). The applied voltage was measured with a 1000:1 passive probe (P6015A, LeCroy, Chestnut Ridge, NY, USA), which was attached to the powered electrode. The total discharge current was calculated by monitoring the voltage drop (Hameg HZ53, Hameg Instruments, Mainz, Germany) in a 6.5 Ω resistance, which was placed between the stainless-steel electrode and the ground. The measurements of applied voltage and the total discharge current were used for the calculation of plasma power [32].

The gases used for the plasma ignition (Ar and compressed air) insert the reactor through the electrode, so that gas bubbles are formed in the liquid. The cubic specimens are placed in the liquid and, depending on their number, are arranged in columns.

In this work, five plasma-enhanced solvolysis experiments were performed by using one, two, three, four, and five cubic specimens in the reactor. The Ar and atmospheric air flows were set to 1 L/min and 2.5 L/min, correspondingly. The plasma power input was initially set to 500 W and adjusted during the process to maintain the solvent temperature at 95 °C for all plasma solvolysis experiments. The time required for the system to reach this temperature was about 20 min after plasma ignition.

The HNO_3_ volume for all dissolution experiments was equal to 200 cm^3^. Periodically, the retrieved carbon fibers were collected, further cleaned in an acetone bath for 10 min, and weighed. Two thermal treatments of the composites under static and agitation conditions were also performed to estimate the plasma effect. In agitation conditions, 200 cm^3^ of HNO_3_ was externally heated (no plasma) to 95 °C, while Ar and atmospheric air flows identical to plasma conditions passed through the solvent-producing bubbles. In static conditions, 200 cm^3^ of HNO_3_ was heated to 95 °C without any solvent stirring. Thermal nitric acid treatments (static and agitation) were applied for the dissolution of one cube, and the retrieval rates were compared to the rates achieved by the plasma-enhanced solvolysis of one cube.

Finally, the recycled fibers were characterized by means of SEM-EDX analysis (JEOL JSM-6300, Peabody, MA, USA). The images were taken using uncoated samples, under high vacuum and an accelerating voltage of 20 kV. The electron beam was focused at different surface areas, and at least five images and EDX measurements were recorded for each sample, to have a better estimation of the retrieved CF surface condition and of the resin removal.

### 2.3. Solvolysis Kinetic Model

**Figure 2:**
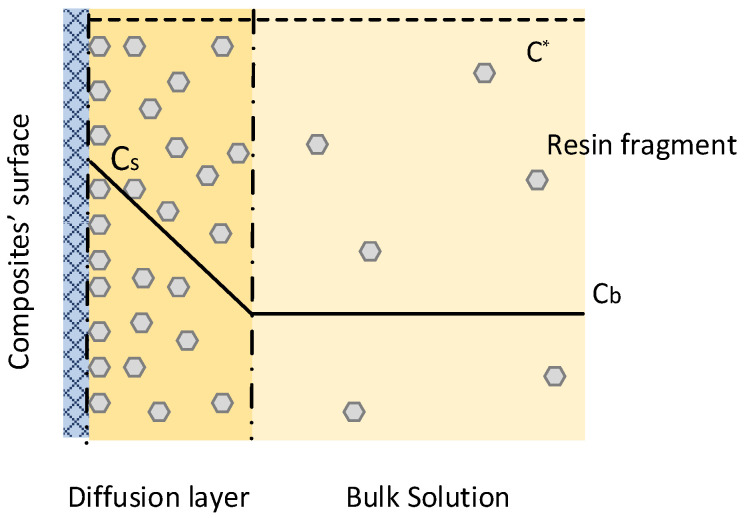
Dissolution kinetics scheme.

The current analysis is based on the dissolution of solid particles as presented in Refs. [36,37,38], with the main assumption that the dissolution of the composites takes place in two consecutive steps. The first step includes disintegration (oxidation in our case) of the polymeric matrix, and as a second step, the produced fragments diffuse from the composite surface to the bulk of the solvent without any change in their chemical structure (Figure 2). If one considers that (a) the main driving forces of this system are the concentration gradients and (b) disintegration and mass transport rates are proportional to the composite surface area *S*, the system is well described by two equations. One is for the disintegration rate $r_{D}$:

(1) $$r_{D}(t)=k_{d}\cdot S(t)\cdot \left(C^{*}-C_{s}(t)\right),$$

One is for the mass transport rate $r_{m}$:

(2) $$r_{m}(t)=k_{SL}\cdot S(t)\cdot {\left(C_{s}(t)-C_{b}(t)\right)}^{n=1},$$

where, $k_{d}$ and $k_{SL}$ are the disintegration and mass transport (solid to liquid) coefficients in cm^3^/cm^2^∙h, S(t) is the composite surface area at time *t* exposed to the solvent of volume *V_sol_* in cm^2^/cm^3^, $C^{*}$ is the solubility of the polymeric matrix under certain conditions (pressure, temperature, shape, and size of composite) in g/cm^3^, $C_{s}(t)$ is the surface concentration of the polymeric fragments in g/cm^3^ at time *t*, $C_{b}(t)$ is the fragment concentration in the bulk of the solvent in g/cm^3^ at time *t*, and *n* is a constant that stands for the number of species that are produced during dissolution of the solute. In our case, we assumed that the polymeric fragments are transported to the bulk without any change in their chemical structure, so *n* was set to 1. It should be noted that $C^{*}$ in the under-investigation system is “apparent” solubility, as nitric acid will decompose and dissolve the polymer matrix, i.e., there will be also chemical reactions rather than simple dissolution.

Moreover, when a pseudo steady state is reached, the rate that the polymeric fragments are produced in the surface will be equal to the rate that they are transported to the bulk of the solvent. This can be expressed as

(3) $$r_{D}=r_{m}=r=\frac{dC_{b}}{dt},$$

A combination of Equations (1)–(3) leads to a rate expression of the form

(4) $$r(t)=\frac{dC_{b}}{dt}=\frac{k_{SL}\cdot k_{d}}{k_{d}+k_{SL}}\cdot S(t)\cdot \left(C^{*}-C_{b}(t)\right),$$

i.e., independent of $C_{s}\left(t\right),$ which is difficult to determine. Equation (4) can be rewritten as

(5) $$\frac{dC_{b}}{dt}=k^{*}\cdot S(t)\cdot \left(C^{*}-C_{b}(t)\right),$$

where

(6) $$k^{*}=\frac{k_{SL}\cdot k_{d}}{k_{d}+k_{SL}}.$$

The resin fragments concentration, *C_b_*, at any time of the solvolysis process can be determined by the amount of recycled carbon fibers that is collected at a specific time, after taking into account the mass content of the composite. In the case of the turbine blade scrap, the resin-to-carbon fiber mass ratio was 0.49:1. Thus, Equation (5) can be written as a function of the mass of the retrieved carbon fibers ($m_{rCF}$):

(7) $$\frac{0.49\cdot dm_{rCF}}{V_{sol}\cdot dt}=k^{*}\cdot S(t)\cdot \left(C^{*}-\frac{0.49}{V_{sol}}\cdot m_{rCF}(t)\right),$$

We prefer to express the rate in terms of the retrieved mass of CFs, as this is the parameter that can be experimentally measured. The composite surface area exposed to the solvent S(t) will drop during the dissolution time and can be also related to the mass of the retrieved fibers that are removed. In our case, where multiple cubic-shaped composites with an edge of length a, are in contact with the solvent, S(t) can be written as

(8) $$S(t)=\frac{s_{comp}(t)}{V_{sol}}=\frac{f\cdot a^{2}(t)}{V_{sol}}=\frac{f\cdot V_{comp}^{\frac{2}{3}}\;(t)\;}{V_{sol}},$$

where $s_{comp}(t)$ is the total surface of the cube in contact with the solvent, *f* is the number of the total faces of the cubes that are in contact with the liquid, and *V_comp_* is the composite volume. It should be noted that Equation (8) results from the experimental observation of the composite shrinking during the dissolution, while its shape remains cubic, i.e., only the cube edge *a*(*t*) decreases. However, the treatment induces roughness and curvatures in the surface, which will lead to higher effective surface area than the one estimated from the flat surface approximation. In the present case of small-size sample dissolution, the underestimation is not important, but a more detailed $s_{comp}\left(t\right)$ function will be necessary to simulate the dissolution of large composite parts.

Moreover, *f* in Equation (8) can and be expressed as follows:

(9) $$f=\sum_{i=1}^{j}\left(4n_{i}+1\right),$$

where *j* is the number of specimens columns and *n_i_* is the number of cubic specimens in column *i*. The number 4 inside the brackets stands for the side faces of each tube, and the number 1 is added to account for the top face of each column. For example, when five cubic specimens are arranged in two columns, four faces of each cube and one top face of each column is in touch with the solvent, giving a total of 22 faces.

In addition, *V_comp_* can be defined by the composite mass (*m_comp_*) and density (*ρ_comp_*, 1.44 g/cm^3^) as follows:

(10) $$V_{comp}(t)=\frac{m_{comp}(t)}{\rho_{comp}}=\frac{m_{p}(t)+m_{CF}(t)}{\rho_{comp}}=\frac{1.49\cdot m_{CF}(t)}{\rho_{comp}}=\frac{1.49\cdot \left(m_{{CF}_{i}}-m_{rCF}(t)\right)}{\rho_{comp}},$$

where $m_{p}\left(t\right),\;m_{CF}\left(t\right),$ are the mass of polymer matrix and the mass of CFs in the composite at time *t*, $m_{rCF}(t)$ is the mass of the retrieved CFs at time *t*, and $m_{{CF}_{i}}$ is the initial CF mass in the composite. The approximation of constant $\rho_{comp}$ is reasonable, as the solvolysis of the specific composite progresses mainly through surface erosion. Sample swelling is negligible even after many hours of immersion in nitric acid, and porosity appears after 3 to 4 h of treatment.

A combination of Equations (7), (8) and (10) leads to the final rate expression:

(11) $$\frac{dm_{rCF}}{dt}=k^{*}\cdot 2.09\cdot f\cdot {\left(m_{{CF}_{i}}-m_{rCF}\left(t\right)\right)}^{2/3}\cdot \left(C^{*}-\frac{0.49}{V_{sol}}\cdot m_{rCF}\left(t\right)\right).$$

In the final Equation (11), solvent volume $V_{sol}$, the initial mass of CFs in the composite $m_{{CF}_{i}},$ and f are process inputs, while the rate $\frac{dm_{rCF}}{dt}$ and the mass of the recovered CFs, $m_{rCF}\left(t\right),$ can be experimentally determined. Thus, Equation (11) can be used for the calculation of the “rate constant coefficient” $k^{*}$ and the solubility $C^{*}$ for all the experimental conditions that are presented below. Finally, it should be noted that Equations (1)–(6) are similar to previous investigations related to dissolution processes [37,38,39] and Equations (7)–(11) were derived in this study to describe the dissolution of the specific composites. In fact, the model can be applied in any dissolution system if one can have an estimation and a mathematical description of the change of the solid shape and size as a function of the dissolution time, i.e., after proper modification of Equation (8).

## 3. Results and Discussion

### 3.1. Experimental Results

**Figure 3:**
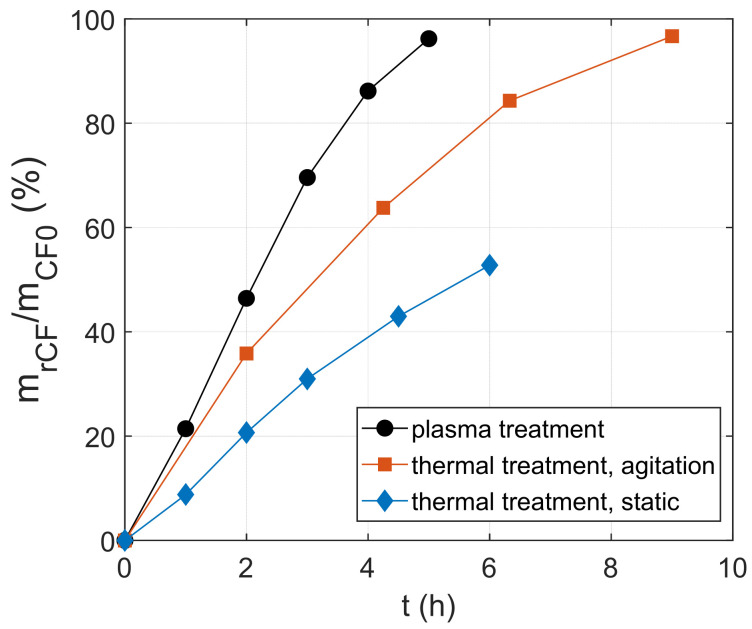
Percentage of mass recovery of CFs as a function of treatment time for plasma-enhanced solvolysis and thermal HNO_3_ solvolysis (stirring and static conditions).

Figure 3 illustrates the % mass retrieval of CFs with plasma and when cubic specimens were thermally treated in warm (95 °C) HNO_3_ (65%) under agitation and static conditions. The volume of treated composites was in all cases ~15 cm^3^. Agitation conditions correspond to heating of the solvent, which is bubbled with gas flows equal to the plasma treatment (Ar 1 L/min and atmospheric air 2.5 L/min). Static conditions correspond to heating of the solvent without any further agitation. The beneficial effect of plasma on the dissolution rate is clear, as the composite was fully dissolved in ~5 h. At the same time, ~70% of the CFs were retrieved from the thermal treated sample under agitation, and only ~40% of the CFs were recovered from the thermal treated sample under static conditions. These results underline the importance of both disintegration and mass transport rate on the total dissolution rate. In the case of plasma, the production of highly reactive species favors the disintegration rate, while the intensive bubbling during the process and the plasma-induced production of ultrasounds and shockwaves also enhance the mass transport rate [32,33,39,40,41,42]. In thermal HNO_3_ treatment under agitation, the mass transport rate is preserved but under a milder oxidative environment; thus, a drop of dissolution rate compared to the plasma treatment is seen. Finally, in the thermal HNO_3_ treatment under static conditions, the mass transport rate is significantly hindered, leading to the lowest dissolution rate.

**Figure 4:**
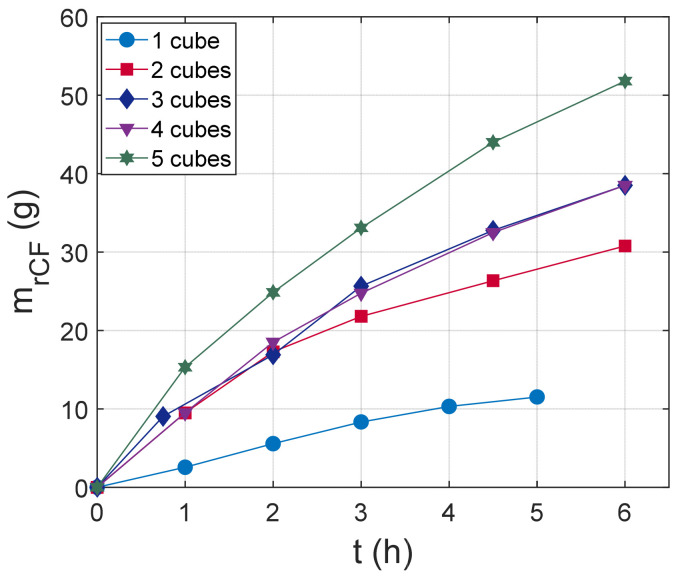
Mass of retrieved CFs as a function of treatment time for 1 cube ($m_{{CF}_{i}}=11.9796\;g)$, 2 cubes $\left(m_{{CF}_{i}}=31.1818\;g\right)$, 3 cubes $\left(m_{{CF}_{i}}=44.1463\;g\right)$, 4 cubes $\left(m_{{CF}_{i}}=57.9818\;g\right),$ and 5 cubes $\left(m_{{CF}_{i}}=68.2127\;g\right)$.

Moreover, five batches with an increasing mass of composite (increasing the number of the cubing specimens placed in the reactor) were plasma-treated under the same conditions (plasma power, final temperature, HNO_3_ volume) for up to 6 h, and the CF retrieval was evaluated. After 1 h or 1.5 h, the process was stopped, and the CFs that were detached from the matrix were collected, cleaned, dried, and weighed. Figure 4 illustrates the recovered CFs’ mass as a function of the treatment time. There is a non-linear increase of the total retrieved CF mass with time for all batches, which indicates a non-constant dissolution rate during the process. In addition, as the number of specimens increases, the total mass of retrieved fibers increases for the specific process time.

For instance, the CF mass retrieved after 3 h from the treatment of 1 cube is 8.3366 g, while from the dissolution of 5 cubes is 33.0734 g. This increase can be partly explained by the increase of the composite surface area exposed to the solvent (*S* in Equation (5)), or in other words, by the higher initial CF mass, as the number of treated cubes increases. Table 1 includes the total initial surface area *S*(*t* = 0), the initial CF mass $m_{{CF}_{i}},$ and the mass of retrieved $m_{rCF}$ after 3 h of treatment. The increase of $m_{rCF}(t=3\;h)$ with either *S*(*t* = 0) or $m_{{CF}_{i}}$ is also not linear, which indicates the complexity of the dissolution mechanism.

**Figure 5:**
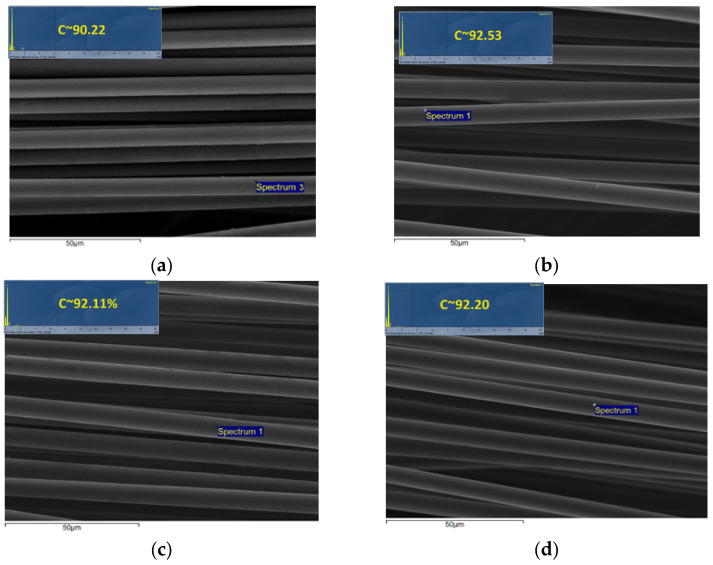
SEM-EDX characterization of rCFs after (**a**) 2 h, (**b**) 3 h, (**c**) 4.5 h, and (**d**) 6 h of plasma-assisted solvolysis of 2 cubes.

It is worth noting that regardless the treatment time or the number of treated cubes, the retrieved fibers were free of resin. Figure 5 presents indicative SEM-EDX images of retrieved CFs from the plasma treatment of 2 cubes after 2 h, 3 h, 4.5 h, and 6 h processing time. SEM images and EDX data were taken for all experimental points of Figure 4, and similar results for the surface condition of the retrieved CFs were obtained. No traces of matrix residuals were observed in the surface; thus, the measured $m_{rCF}$ reflects exclusively the mass of CFs. Finally, the % carbon content as estimated from EDX measurements is close to virgin fiber (~92%), which indicates that no significant damage from surface oxidation was induced by plasma treatment. The small difference of % C content between virgin and retrieved CFs was also reported in Ref. [34], where more accurate XPS analysis was used.

### 3.2. Kinetic Model Results

**Figure 6:**
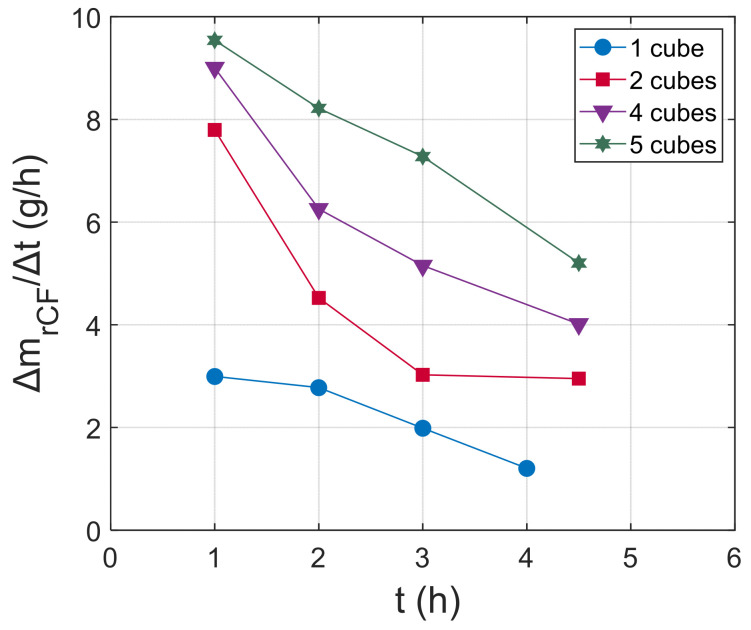
Experimental data of CF retrieval rate as a function of process time for the dissolution of 1, 2, 4, and 5 cubes.

Figure 6 illustrates the experimental data of the CF retrieval rate $\frac{\Delta m_{rCF}}{\Delta t}\;(\frac{g}{h})$ as a function of the process time for the plasma treatment of 1, 2, 4, and 5 cubic specimens. The experimental results show that the retrieval rate decreases with the process time for all cases. The non-constant retrieval rate was already predicted from Figure 4, and the drop with process time can be explained after taking into account the terms in Equation (5). The rate $\frac{\Delta m_{rCF}}{\Delta t}$ will be a function of the surface-to-solvent volume ratio S(t) and the mass concentration gradient $\left(C^{*}-C_{b}(t)\right)$. S(t) will drop with process time, as the composite surface in contact with the solvent is reduced during dissolution, and the same is true for the concentration gradient $\left(C^{*}-C_{b}(t)\right)$, as higher amounts of the polymer matrix fragments will be transported to the liquid phase with time. It is also worth noting that the highest rates are always observed at the initial stages of the dissolution; thus, an efficient strategy for achieving high rates during the whole process can be (a) the addition of composite mass during the process to maintain a high value of $S\left(t\right)$ and (b) the use of excess solvent volume $\left(V_{sol}\right)$ to realize a negligible drop of the concentration gradient. It should be noted that (a) can be applied when the target is the retrieval of short CFs and the components for recycling can be cut in different sizes and shapes. In this case, the solvolysis of the next component can start before the full dissolution of the previous one, thus maintaining high retrieval rates and high process throughput.

Equation (11) was then fitted to the experimental data for CF retrieval rate (Figure 6) by minimizing the sum of the squared residuals. The initial fitting tests show that the apparent solubility $C^{*}$ remains constant for all the experiments, so the fitting parameter was the “rate constant coefficient” $k^{*}$. Constant solubility $C^{*}$ is reasonable, as it depends only on the chemistry of the solute, the solvent, the temperature, and the pressure, which are all the same in these experiments. The experimental data for t ≥ 1 h were used for the fitting to ensure constant solution temperature. In plasma enhanced solvolysis, plasma power is responsible for the heating of the solution, and for the specific set of conditions (plasma power, solvent volume), a steady temperature of 95 °C is reached after 20 min. Both $k^{*}$ and $C^{*}$ will be affected by temperature; thus, the fitting was applied to the process conditions where the temperature is constant.

**Figure 7:**
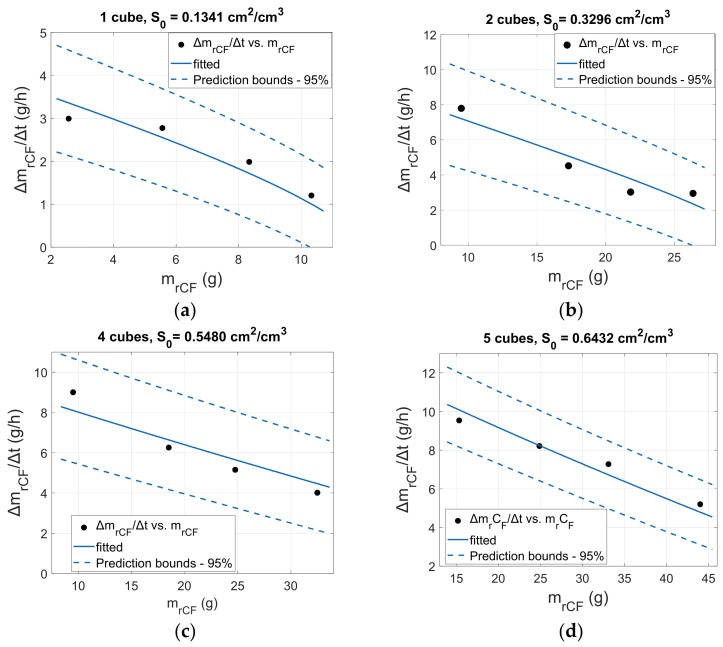
Experimental and fitted values of the CF mass retrieval rate for (**a**) 1 cube, (**b**) 2 cubes, (**c**) 4 cubes, and (**d**) 5 cubes.

Figure 7 illustrates the experimental data and the fitted curves of the CF retrieval rate $\frac{\Delta m_{rCF}}{\Delta t}\;(\frac{g}{h})$ as a function of the total retrieved CF mass ($m_{rCF})$ for the plasma treatment of one, two, four, and five cubic specimens. In addition, Figures S1, S2 and S3 present the experimental data and the fitted curves for the thermal treatment under static conditions, the thermal treatment under agitation, and the plasma treatment of three cubes, respectively.

The deviation between the predicted and experimental values of $\frac{\Delta m_{rCF}}{\Delta t}$ lies between 5 and 20% for all the experimental sets, which allows us to express that the general scheme we adopted for the process can well describe the dissolution mechanism. The higher deviation is observed for larger treatment times, which is attributed to the constant composite density assumption and the consequently less accurate calculation of *S*(*t*) from Equations (8) and (10). At the later dissolution stages, the shape of the composite is also irregular, and the CF-to-matrix mass ratio drops; this may lead to further errors in the calculation of *S*(*t*). Table 2 summarizes the predicted k* for the different experiments while the best fitting results were obtained when the resin fragments’ solubility C* was set to 0.3886 g/cm^3^. It is noticeable that the predicted value of k* is not constant and drops with the number of cubes, i.e., with the initial mass of the composite. The calculation of the epoxy matrix solubility C* is extremely important as it defines the minimum volume of solvent that is required for the dissolution of the composite and is also important for the design and construction of the solvolysis reactors. In addition, the calculation of k* provides valuable information for the total dissolution rate and the rate limiting steps.

According to Equation (6), the value of $k^{*}$ depends on the disintegration $k_{d}$ and mass transport $k_{SL}$ coefficients, and the value of k* will be determined from the rate limiting step. If the $k_{SL}\ll k_{d}$, then $k^{*}\sim k_{SL},$ while in the opposite case, $k^{*}\sim k_{d}$.

Based on the literature findings, a hard estimation of both $k_{d}$ and $k_{SL}$ can be performed, to understand the parameter that determines the dissolution rate. The disintegration of polymer matrices in HNO_3_ epoxy-based composites progress via oxidation of either ester bonds in the case of anhydride hardeners or oxidation of amino bonds in the case of amine hardeners [28,29,43]. The species responsible for the oxidation can be either NO_2_/NO_3_ species from the thermal decomposition of concentrated HNO_3_ [28,30,44] or plasma-produced oxygen-containing species such as ·OH or ·O [32,33]. The oxidation rate constants and their Arrhenius expressions for such reactions can be found in detailed organic chemistry databases [44,45]. For the NO_3_ oxidation of ester bonds at 95 °C, the calculated rate constant is $k_{oxe,{NO}_{3}}=3.3\cdot 10^{-15}\;{cm}^{3}/molec\cdot s$, while the value for ·OH oxidation is $k_{oxe,OH}=2.5\cdot 10^{-12}\;{cm}^{3}/molec\cdot s$. The corresponding rate constant values for the oxidation of amine bonds are $k_{oxa,{NO}_{3}}=3.2\cdot 10^{-14}\;{cm}^{3}/molec\cdot s$ for NO_3_ and $k_{oxa,OH}=1.2\cdot 10^{-11}\;{cm}^{3}/molec\cdot s$. Oxidation rate constants can be correlated to the disintegration rate coefficient $k_{d}$ if they are multiplied by the number of polymer molecules per cm^2^ of the composite ($N_{s})$. This number can be calculated from the mass density of the composite (1.44 g/cm^3^), the mass fraction of the polymer matrix to the composite (0.33), and after assuming an average molecular weight for the polymer matrix (${MW}_{m}$) and the surface thickness (d). For typical values, ${MW}_{m}\sim 1000\;g/mol$ and $d=1\cdot 10^{-7}\;cm$, $N_{s}$ can be calculated as

(12) $$N_{s}=\frac{{0.33\cdot \rho }_{comp}\cdot d\cdot N_{A}}{{MW}_{m}}=2.8\cdot 10^{13}\;molec/{cm}^{2},$$

where $N_{A}$ is the Avogardo number.

An estimation of the disintegration rate $k_{d}$ can then be performed for the worst-case scenario of the oxidation of ester bonds by NO_3_ species (lower rate constant) as

(13) $$k_{d}=k_{oxe,{NO}_{3}}\cdot N_{s}\cdot 3600\;\left(s/h\right)=3.5\cdot 10^{2}\;{cm}^{3}/{cm}^{2}\cdot h$$

The calculated value of the $k_{d}$ is much higher compared to all the values of k*, indicating that oxidation and matrix disintegration is probably not the rate-limiting step of the dissolution process.

As for the mass transport coefficient $k_{SL}$, there are no reported values for the solvolysis of composites. However, the mechanisms of mass transport from the solid to the liquid phase during the dissolution of polymers and resins has been the subject of several studies [46,47,48,49,50]. Diffusion is considered as the main transport mechanism, but convective mass transport cannot be ignored, especially in cases where intense agitation is applied and turbulence intensity is rather high. Pressure, temperature, vessel shape and dimensions, solvent viscosity, agitation speed, and solid volume to liquid volume ($C_{v}$) are the parameters that will most affect the mass transport coefficient $k_{SL}$. The effect of $C_{v}$ on $k_{SL}$ is of specific interest in this study, as our kinetic experiments were performed by varying the composite volume while the solvent volume remained the same. In Ref. [50], a detailed analysis of the effect of $C_{v}$ on $k_{SL}$ was presented, and although the dissolution process is not the same (dissolution of ion exchange resin from NaOH solution), the results of the investigation are in good agreement with the present findings. The calculated values of $k_{SL}$ are between 0.01 and 35.0 cm^3^/cm^2^∙h, i.e., within the range of $k^{*}$ that was calculated in our experiments. Similar values of $k_{SL}$ have been reported in several studies for different processes and different agitation methods (impellers, spargers) [51,52,53]. It is also interesting that according to Ref. [51], the increase of the solid-to-liquid volume ($C_{v}$) results in an optimum of $k_{SL}$ at $C_{v}=0.2$; after that, a significant drop of $k_{SL}$ was measured. The variation of $k_{SL}$ with $C_{v}$ is related to changes in the apparent viscosity, which in general increases with $C_{v}$ [54]. The decrease in $k^{*}$ with $C_{v}$ is also predicted from our calculations, and together with the similar values of $k^{*}$ and reported $k_{SL},$ provide strong indications that under the present conditions the composite dissolution rate is limited by mass transport. It is also worth noting that in our experiments where multiple specimens were dissolved, the shielding effects between adjacent cubes were of significant importance and further hindered mass transport of resin fragments. Thus, further improvements of the dissolution rate should be focused on more efficient mass transport. Static and swelling experiments at different temperatures are in progress in order to estimate the diffusion coefficient in the specific system, while different gas flows and agitation methods (sonication, magnetic stirring) should be tested to estimate and improve the convective transport term.

## 4. Conclusions

In the current work, an investigation of the dissolution kinetics in the conventional nitric acid and plasma-enhanced nitric acid solvolysis of CFRPs was performed. Plasma was found to significantly increase the dissolution rate due to the enhancement of both the oxidation and mass transport rate of the polymer matrix.

The increase of the CFRPs’ mass while the solvent volume remained constant also resulted in an increase of the dissolution rate, mainly due to the higher surface of the composite in contact with the solvent. In all experiments, the dissolution rate is not constant and drops with process time because of the composite surface reduction and the drop of the concentration gradient between polymer matrix fragments in the composite surface and the solution. The addition of composite mass during the process and the use of excess solvent can be used to maintain high dissolution rates during the whole process.

The implementation of the dissolution kinetic model and the fitting of the experimental results of CF retrieval rate led to the calculation of the polymer matrix solubility of the plasma-enhanced nitric acid solution and the process total rate coefficient. The rate coefficient was further analyzed for the reaction (disintegration) rate and the mass transport (solid to liquid) rate coefficient. Based on the literature findings, the total rate coefficient is determined from the mass transport coefficient, which for the specific set of experiments is the most probable dissolution rate-limiting factor.

## Figures and Tables

**Table 1:** Initial surface area *S*(*t* = 0), initial CF mass $m_{{CF}_{i}}$, retrieved CF mass $m_{rCF}\;(t=3\;h)$ after 3 h of plasma treatment, and standard deviations of these values for different numbers of specimens.

Number of Cubic Specimens	Total Initial Surface Area *S*(*t* = 0) (cm^2^/cm^3^)	$\mathrm{Initial}\;\mathrm{CF}\;\mathrm{Mass}\;m_{{CF}_{i}}$ (g)	Retrieved CF Mass $m_{rCF}(t\;=\;3\;h)$ (g)
1	0.1341 ± 0.0042	11.9796 ± 0.0003	8.3366 ± 0.0005
2	0.3196 ± 0.0180	31.1818 ± 0.0004	21.8050 ± 0.0003
3	0.4305 ± 0.0344	44.1463 ± 0.0005	25.6391 ± 0.0006
4	0.5480 ± 0.0547	57.9818 ± 0.0005	24.7574 ± 0.0005
5	0.6432 ± 0.0828	68.2127 ± 0.0006	33.0734 ± 0.0007

**Table 2:** Calculated k* for different experimental conditions and solid-to-liquid volume $C_{v}$.

Number of Cubic Specimens	k* (cm^3^/cm^2^h)	$C_{v}\;({cm}^{3}/{cm}^{3})$
1	0.1887	0.06
2	0.1213	0.16
3	0.0855	0.23
4	0.0444	0.30
5	0.0443	0.35

## Data Availability

The original contributions presented in this study are included in the article. Further inquiries can be directed to the corresponding author.

## Supplementary Materials

The following supporting information can be downloaded at: https://www.mdpi.com/article/10.3390/ma18184242/s1, Figure S1: Experimental and fitted values of the CFs’ mass retrieval rate for the thermal treatment experiment under static conditions; Figure S2: Experimental and fitted values of the CFs’ mass retrieval rate for the thermal treatment experiment under agitation conditions; Figure S3: Experimental and fitted values of the CFs’ mass retrieval rate for the 3 cube dissolution experiment; Table S1: Calculated *k** for the thermal treatment under static conditions, thermal treatment under agitation and plasma treatment of one cube together with the corresponding values of solid to liquid volume $C_{v}$.
