# Supplementary material for: Dissolution Kinetics in Plasma-Enhanced Nitric Acid Solvolysis of CFRCs
Source: Materials (Basel). 2025 Sep 10;18(18):4242. doi: 10.3390/ma18184242 (PMC12471681; doi:10.3390/ma18184242)
Supplement: Supplementary file 1 [file materials-18-04242-s001.zip › materials-3814856-supplementary.pdf]

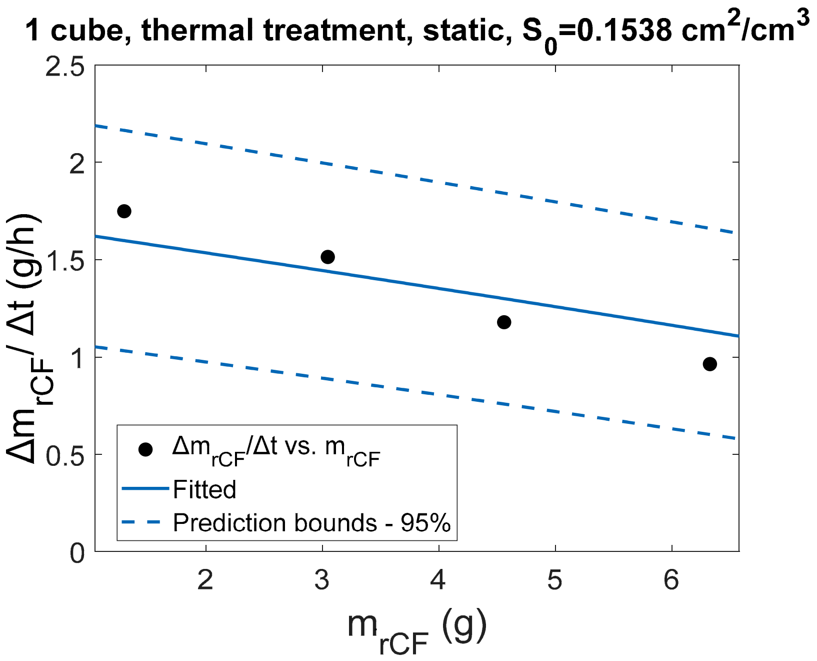

Figure S1: Experimental and fitted values of the CFs' mass retrieval rate for the thermal treatment experiment under static conditions.

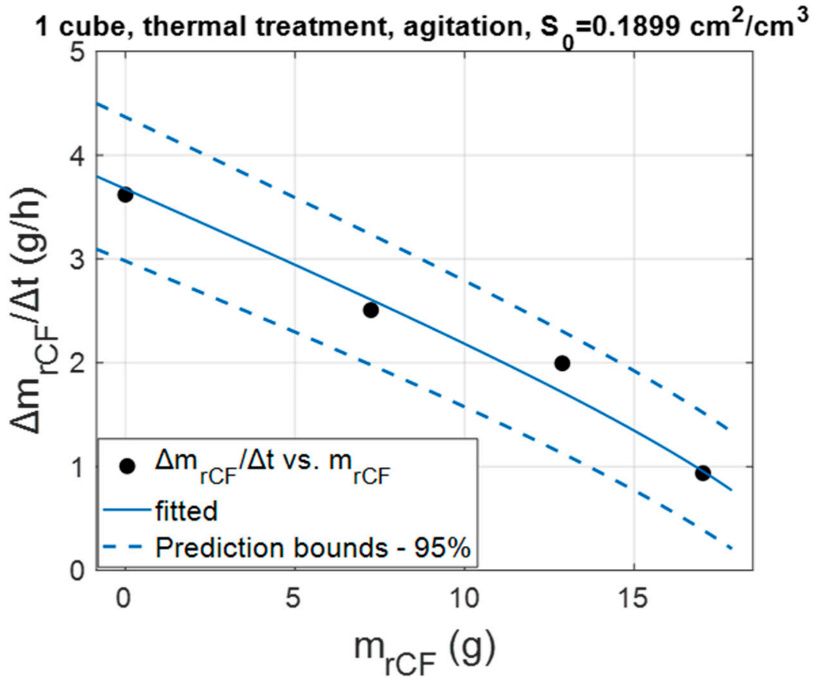

Figure S2: Experimental and fitted values of the CFs' mass retrieval rate for the thermal treatment experiment under agitation conditions.

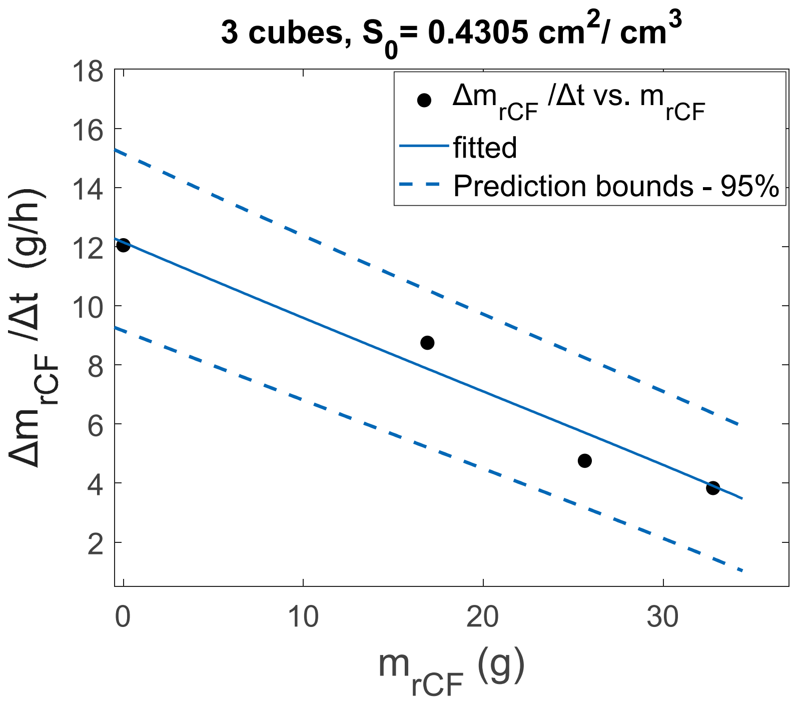

Figure S3: Experimental and fitted values of the CFs' mass retrieval rate for the 3 cube dissolution experiment.

Table S1: Calculated k^{*} for the thermal treatment under static conditions, thermal treatment under agitation and plasma treatment of one cube together with the corresponding values of solid to liquid volume C_v.

**Number of cubic specimens**	k^{*} **(cm³/(cm²h)**	C_v **(cm³/cm³)**
1 (thermal treatment, static)	0.0702	0.058
1 (thermal treatment, agitation)	0.1220	0.063
1 (plasma)	0.1887	0.060
